# Midwives **‘**views of parents’ questions and expectations on prenatal genetic testing - identifying informational needs in prenatal genetic counselling

**DOI:** 10.1007/s12687-025-00846-8

**Published:** 2025-12-13

**Authors:** Lisa Åkerman, Maria Johansson Soller, Charlotta Ingvoldstad Malmgren

**Affiliations:** 1 Department of Clinical Genetics, Karolinska University Hospital, Stockholm, Sweden; 2 Molecular Medicine and Surgery, Karolinska Institutet, Stockholm, Sweden; 3 Center for Fetal Medicine, Karolinska University Hospital, Stockholm, Sweden

**Keywords:** Information, Informed choice, Genetic counselling, Decision making, Prenatal diagnosis, Chromosome aberrations

## Abstract

Autonomy and informed decision making are important aspects for prenatal genetic screening and diagnostics. Midwives’ knowledge and skills are essential to provide adequate information about prenatal testing to expecting parents to enable informed decisions. Information from midwives to parents about prenatal genetic testing has been found to not always be adequate, and parents’ needs not always understood.​ As new methods are introduced, the scope of analysis is widening. In order to achieve informed decision-making, it is important to understand the questions and expectations midwives meet from expecting parents. This study explores the questions and expectations midwives meet from expecting parents regarding prenatal genetic testing, and how uncertainties are perceived and valued. A questionnaire was distributed through a midwife with a national coordinating role, to all midwives in primary maternity care and to the 8 ultrasonography clinics in the Stockholm region, as well as to midwives across Sweden via regional coordination midwives. The responding midwives (*N* = 71) represented different health care regions in Sweden, working both in primary maternity healthcare and as ultrasonography specialists. Midwives were found to perceive an increased number of questions about noninvasive prenatal testing (NIPT) but a proportion of midwives are not completely confident to answer these questions. Midwives get questions about trisomy 21, other trisomies and sex chromosome abnormalities, but also neuropsychiatric conditions. Methods for invasive, diagnostic testing do not seem to be discussed when accepting offer of initial screening. ​ Midwives are aware of uncertain and secondary findings, but fewer have discussed this with parents. Continuing education and support for midwives is essential – and should put additional focus on developing understanding around established methods like NIPT, but also on more comprehensive genomic test methods such as microarray and massive parallel sequencing techniques as well as challenges around discussing conditions tested for and test results, including uncertain results and secondary findings, with expecting parents.

**Supplementary information:**

The online version contains supplementary material available at 10.1007/s12687-025-00846-8.

## Introduction

One aim of a generally offered prenatal screening program is to achieve reproductive autonomy. Essential for adequate information is that it is easy to understand, addresses key benefits, limitations and risks, and is delivered in a non-directive manner, so that expecting parents can make informed reproductive decisions (Dondorp et al. 2015; Sahin-Hodoglugil et al. 2023; SMER 2012, 2015). To be able to make an informed decision whether to accept or decline prenatal testing, there is a need for expecting parents to understand the screening options, potential outcomes and consequences of both the prenatal testing results and conditions tested before making any decisions.

Receiving prenatal test results indicating that the expected child might have an anomaly can be a complex and emotionally challenging process (Kernie et al. 2022; Lou et al. 2020). In a limited timeframe for further diagnostics and options for the pregnancy, rapid decisions that can be life changing are needed. The results and possible genetic findings during pregnancy can help the emotional preparation for a life with a child with a condition or can support the informed decision of termination of the pregnancy. The information on the condition can also lead to more adequate medical management and preparations from a healthcare perspective during and after pregnancy (Sahin-Hodoglugil et al. 2023).

In Sweden, the offer of prenatal testing methods varies across the 21 healthcare regions as all regions independently decide what to offer. In most of the regions, First Trimester Combined test (FCT) is offered either to all pregnant women or to women over 35 years, but three regions do not offer this at all. In most regions NIPT is offered as a second-tier test if the FTC indicates a high probability result for a trisomy. Non-invasive prenatal test (NIPT) is instead of FCT offered as the first-tier test in two regions, and in some regions NIPT is also offered through private actors for those who do not fulfill the criteria for the NIPT offer in their own region. The screening offered by the regional health care providers is free of charge, NIPT through private actors for those who do not fulfill the criteria are paid by the patient. Around 4% of pregnancies are identified by FCT as having an > 1/200 probability for an anomaly, which in most counties in Sweden, is the threshold for further testing for an anomaly. If an increased probability is identified by FCT, most women (94% in 2021) continue with the offered testing methods (NIPT, chorion villus biopsy (CVB) or amniocentesis (AC) (Skogsdal et al. 2023). If NIPT indicates a high probability for a trisomy, an invasive testing is recommended.

Within the maternity care system in Sweden, midwives serve as the main point of contact for all pregnant woman and they have an independent responsibility for normal pregnancies (Barnmorskeförbundet 2022). Midwives in primary maternity healthcare are therefore also responsible for providing the initial general information on the offer of prenatal testing and are often the ones referring to the FCT testing procedure. According to the Swedish law of genetic integrity, all pregnant women should be offered general information about prenatal screening (Riksdagen 2006). It is also stated in the directive guidelines of the Swedish national board of health and welfare (Socialstyrelsen, 2012) that this general information should be provided by the healthcare professionals at the outpatient maternal care clinic. Thus, midwives have an essential role in understanding the needs of expecting parents, providing information in a non-directive way, and setting realistic expectations– thereby also having a key role in supporting informed decision-making from the woman. The midwives follow the women throughout the pregnancy, even if they have been referred to a specialist center for follow-up diagnostics. Therefore, they might need to discuss for example uncertain findings and invasive testing. Some midwives work at specialized ultrasonography clinics/settings or at fetal medicine units at hospitals, and some are certified to perform the FTC ultrasonography (Carlsson et al. 2021). The formal education in genetics is still limited, but there are regional initiatives covering newer methods (personal communication, C Malmgren Ingvoldstad).

Previous studies show that the parents’ expectations, questions and values regarding prenatal testing are not always well understood by midwives, and maternal healthcare is not always able to fulfill their informational needs (Carlsson et al. 2021; Ferm Widlund et al. 2009; Skogsdal et al. 2023; Ternby et al. 2015) and therefore decisions about prenatal screening are not always well informed (Di Mattei et al. 2021; van den Berg et al. 2006). As new genetic testing methods are implemented into clinical use and the prenatal testing options are becoming more complex, it is important to build understanding of the informational needs among maternity healthcare specialists.

One of the concerns with new screening methods, such as NIPT, is that an easier procedure can be perceived as a near risk-free routine test and no increased risk of miscarriage, and would therefore not require much pre-test counselling. Absence of pre-test counselling can lead to that parents might undergo the testing without reflecting and without understanding the possible consequences of the test– thus not achieving an autonomous informed reproductive choice (Dondorp et al. 2015; SBU 2015; SMER 2015). The information of the high level of accuracy of NIPT-results, compared to FTC, sometimes makes the NIPT-test wrongly thought of as a diagnostic test.

Newer, more accurate and comprehensive prenatal analysis methods tests with the possibilities to analyze the whole genome also have ethical concerns and implications for counselling Harris et al. (2028), Harris et al. (2018). One concern for counselling is the possibility of uncertain results. New sequencing methods that explore large panels of genes in one test are increasingly being used in prenatal applications (Liu and Vossaert 2022; Mellis et al. 2022a; Mellis, Tapon, Mellis et al. 2022a, b; Sveriges Regioner i Samverkan, 2022) and while this brings increased diagnostic resolution, it will at the same time bring a higher likelihood of an uncertain result. Uncertainties include incomplete knowledge of gene-disease correlation, how a genetic anomaly is presented prenatally, the pathogenicity of an anomaly, and variants of uncertain significance (VUS). Also, unexpected results could arise in form of secondary findings (SFs) or incidental findings (IFs), i.e. finding a genetic change that might have implications for other members in the family, but is not related to the findings identified in the fetus (Klapwijk et al. 2021). Uncertain results can make the decision-making process even more difficult to parents, and have an emotional impact, including decisional regret, shock, and worry (Harding et al. 2020). Thus, to achieve an informed decision about genetic prenatal testing, it is important for expecting parents to be aware of the possibility of uncertain results and secondary findings with the more comprehensive methods.

This study explores midwives’ views of the interaction with expecting parents regarding prenatal genetic testing. The overall aim of this study is to build understanding of the questions midwives meet from expecting parents regarding prenatal genetic testing, considering new possibilities of analysis, and their confidence to answer these questions. Furthermore, the study explores how results containing uncertainties or secondary findings is perceived by midwives.

## Methods

### Study design, material and method

The study is a descriptive, cross-sectional study with a quantitative methodological approach. A web-based questionnaire was developed specifically for this study. The questionnaire is composed of 14 survey questions, followed by 8 demographic questions. The survey includes questions about the expectations midwives meet from parents around prenatal diagnosis and whether these have changed over the last 3 years, and also questions on what methods and diseases/syndromes are discussed with expecting parents and what parents’ reasons are perceived to be for considering prenatal diagnostics. Furthermore, the survey includes questions about uncertain results and secondary findings. Respondents were presented with short texts explaining uncertain findings and secondary findings (SFs), and asked questions about awareness, and perceived knowledge, of these concepts, and aspects of reporting them to expecting parents. For all questions there was an optional possibility to leave a free-text comment. The questionnaire was developed by a genetic counselor student in collaboration with two experienced experts in genetic counselling. The development was done in line with previous studies and literature. The questionnaire was pretested with 3 genetic counselling students, and subsequently with 8 midwives during an educational event. The midwives in the pretest included both primary maternity care and ultrasonography midwives, from different health care regions in Sweden. Based on feedback a few of the questions were modified for clarity. The questionnaire is constructed in the online tool Survey & Report. The complete questionnaire is given in Supplementary 1.

### Data collection methods, survey administration and data analysis

The survey was distributed between October and November 2022. A link to the survey, together with an informational letter, was distributed through a midwife with a national coordinating role to all midwives in primary maternity care in the Stockholm region (440 midwives from 70 clinics). Additionally, it was distributed to the management of 8 ultrasonography clinics in Stockholm and regional coordination midwives across Sweden, with a request to distribute in their respective organizations. Inclusion criteria were to be working as a midwife in primary maternity care or in a specialized clinic, within that group there were no exclusion criteria. The questions in the survey with pre-defined alternatives and the yes/no answer questions were subject to descriptive statistics. Means and standard deviations were calculated for rating scale-type questions, in Excel and IBM SPSS Statistics (Version 28).

### Ethical considerations

According to what is stated in the Ethical Review Act (Riksdagen 2003), this study does not require permission through Swedish Ethical Review Authority (Etikprövningsmyndigheten, 2022). The study is planned and carried out in accordance with *Good Research Practice* (*God Forskningssed*, 2017) and in line with the guidelines of the Declaration of Helsinki (WMA 2013). In the introductory information it is stated that participation is voluntary, and that submitting the survey is considered as an informed consent.

## Results

### Demographic characteristics

A total of 71 midwives responded to the questionnaire; both maternity healthcare midwives (*n* = 56) and ultrasonography midwives (*n* = 18), working either in public (*n* = 57) or private clinics (*n* = 14). Almost half (*n* = 33) of the midwives had partaken in genetic education in the past 3 years. The sociodemographic characteristic of participants is presented in Table 1.

**Table 1:** Sociodemographic characteristics of participants

*N* = 71,* n (*%)			
Role	Maternity healthcare midwife	56	(79)
	Ultrasonography midwife	13	(18)
	Other	2	(3)
Age	< 35	7	(10)
	35–50	34	(48)
	> 50	30	(42)
Years in profession	0–4 5–15 16–25 > 25	10 31 18 12	(14) (44) (25) (17)
Clinic type	Public Private	57 14	(80) (20)
Area (as asked to respondents)	Medium sized city Smaller town	39 19	(55) (27)
	Larger city	13	(18)
Partaken in genetic education past 3 years	Yes	33	(46)
	No	36	(51)
	No answer	2	(3)

### General information and questions about prenatal genetic testing

Most midwives discuss prenatal genetic testing, at some level, with the expecting parents they meet in early stage of the pregnancy; 75% (*n* = 53) state they discuss this with 8–10 out of 10 of couples they meet. The most common reasons for the midwives not to discuss prenatal diagnostics with parents are that parents already received this information elsewhere (*n* = 29, 40%) and that parents choose not to receive this information (*n* = 8, 11%). As a reason for not discussing testing, none of the midwives stated lack of time and only 1% (*n* = 1) lack of knowledge. More than half of the midwives (*n* = 46, 65%) stated that few (0–2 out of 10) parents ask additional questions about genetic prenatal testing, and that the overall number of questions from expecting parents have stayed either the same (*n* = 41, 58%) or have increased (*n* = 30, 42%) over the past three years. None of the midwives stated the number of questions had decreased.

### Questions on specific conditions

**Fig. 1:**
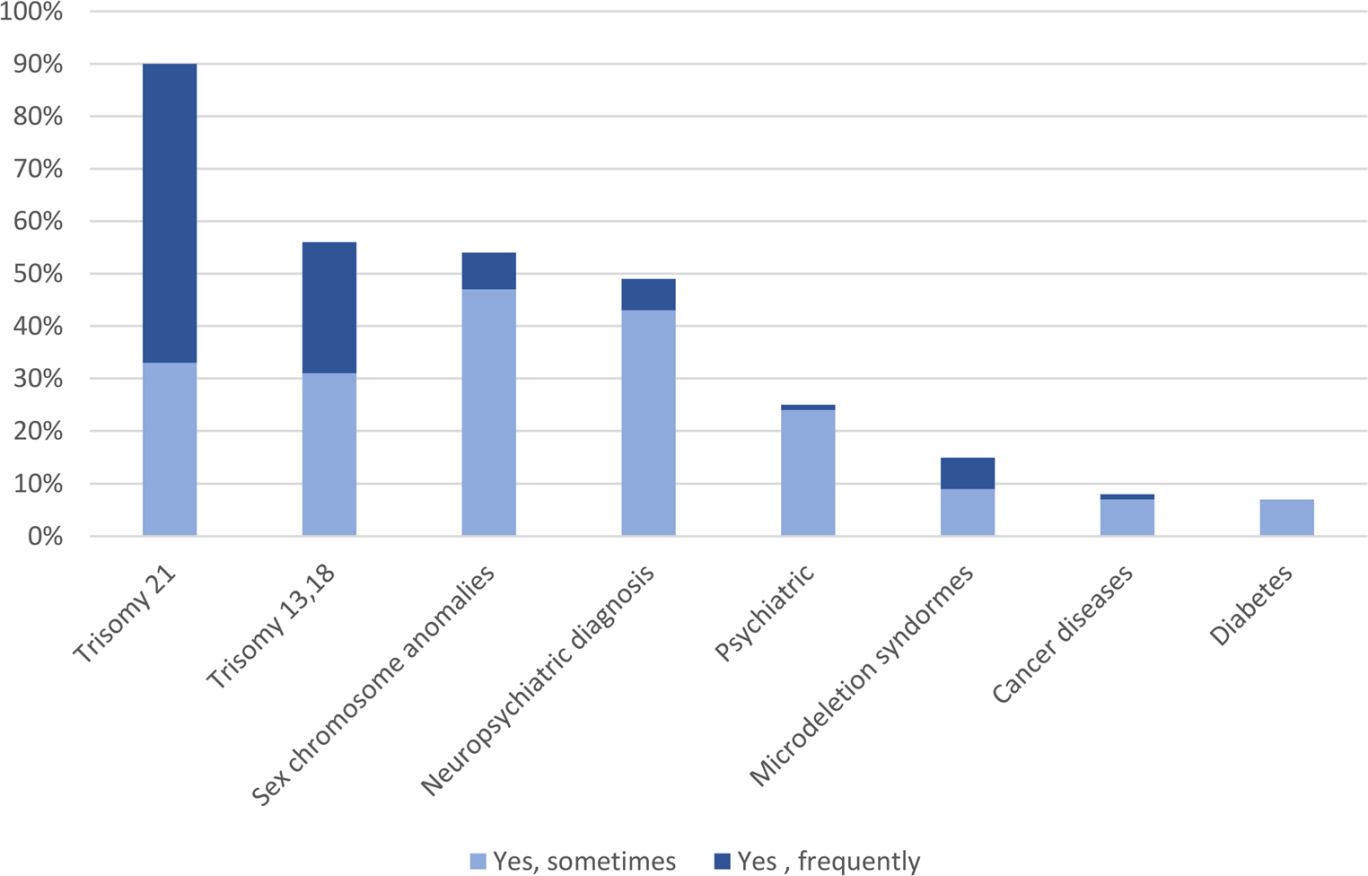
Questions about specific conditions. Note. Midwives were asked if they get questions from parents on different diseases/syndromes, with the alternatives “No, I have not”, “Yes, rarely-sometimes”, “Yes, regularly-frequently”. In the figure the two latter options are displayed

When asked if the parents ask questions about specific conditions, most midwives (*n* = 63, 90%) indicated that they frequently or sometimes get questions about testing for Trisomy 21 from expecting parents, less frequently about Trisomy 13/18 (*n* = 40, 56%), sex chromosome anomalies (*n* = 38, 54%) and neuropsychiatric diagnosis (*n* = 34, 49%). Other types of conditions specified in the question - psychiatric diagnosis, microdeletion syndromes, cancers, diabetes - were also, but less frequently, indicated as subject of questions (Fig. 1). In addition to the specified disorders in the question, free text comments mention heart conditions, muscular dystrophy, cystic fibrosis, metabolic diseases, among others. Furthermore, comments indicate that specific conditions present in the family were the cause of questions from expecting parents.

### Questions on methods and confidence to explain it

**Fig. 2:**
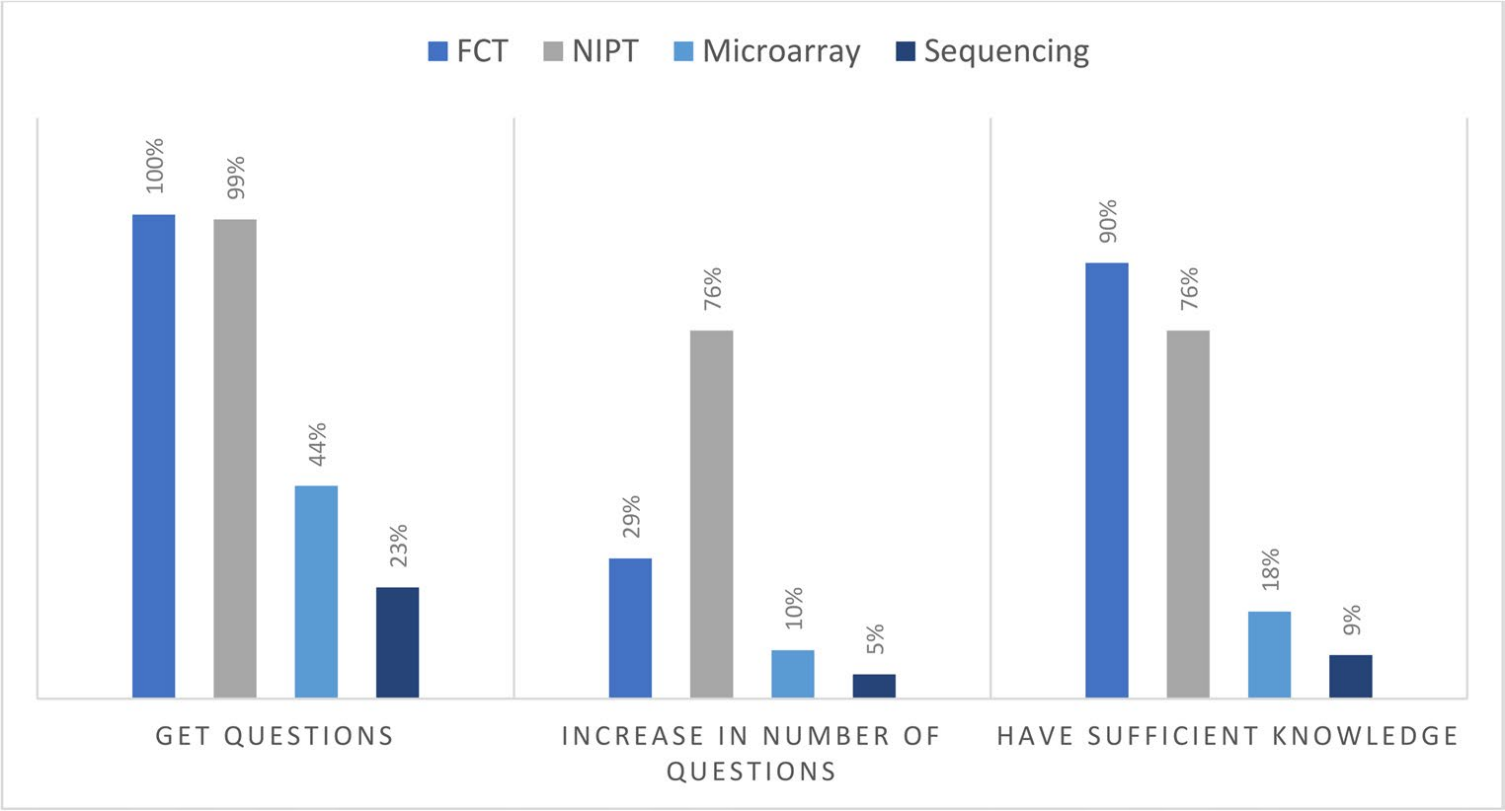
Questions and knowledge about methods. Note. Midwives were asked if they get questions about methods with alternatives “Never”, “Rarely-sometimes” and “Regularly-frequently”, and if number of questions have changed in the past three years, with the alternatives “Increased”, “No change” and “Decreased”. They were also asked if they perceive themselves to have sufficient knowledge to explain and answer questions with the alternatives “No”, “Partly” and “Yes”. The options of “Rarely-sometimes” and “Regularly-frequently” are combined in this diagram

Respondents were asked whether they receive questions from expecting parents about FCT, NIPT, microarray and sequencing methods with the alternatives “No, I have not”, “Yes, rarely-sometimes”, “Yes, regularly-frequently” (Fig. 2). Almost all responding midwives (*n* = 67, 100% and *n* = 66, 99%, respectively) are asked about FCT and NIPT to some extent (the two options “Regularly-frequently” and “Rarely-sometimes” combined). Questions about invasive sample analysis methods are less frequent; 44% (*n* = 30) of midwives indicated they get questions about microarray and 23% (*n* = 16) about sequencing methods. Respondents were also asked if they noticed a change in the type and number of questions in the past three years. 69% (*n* = 36) of midwives perceive that the number of questions about FCT have not changed over the past three years, while 29% perceive an increase in number of questions. For NIPT, 76% (*n* = 37) of midwives perceive an increase, and 22% (*n* = 11) no change in number of questions. For microarray and sequencing, most midwives (*n* = 36, 90% and *n* = 36, 92% respectively) do not perceive a change in the number of questions the past three years. Respondents were further asked if they feel they have enough knowledge to explain and answer questions about the methods (Fig. 2). Most midwives stated to have sufficient knowledge about FCT (*n* = 63, 90%) and NIPT (*n* = 53, 76%). Midwives stated to be less knowledgeable about genetic analysis methods related to invasive samples; 19% (*n* = 13) state to have enough knowledge about microarray, and 9% (*n* = 6) about sequencing methods.

### Reasons for testing and value of test attributes

Respondents were asked to rate expecting parent´s reasons for testing. Four different reasons were evaluated on a 5-point scale. Highest rated by midwives was the general statement “To test as much as possible – know everything is ok” (mean 3,5), followed by “Worry about condition in the family” (mean 2,48), “Worry about a rare, serious disease” (mean 1,87) and the lowest rated statement “Worry about a common adult-onset disease” (mean 1,07).

### Secondary findings and uncertain findings

**Fig. 3:**
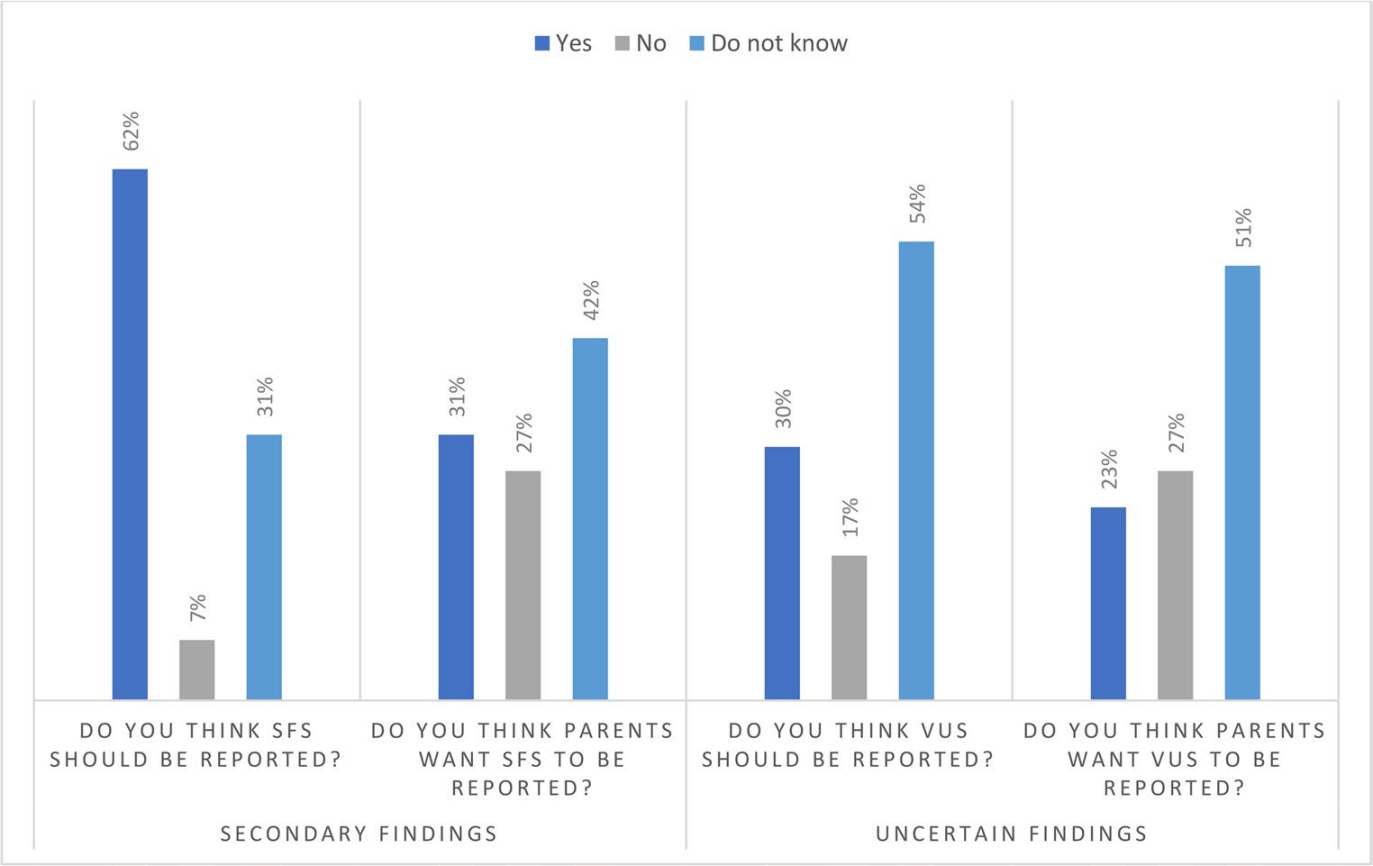
Reporting of secondary and uncertain findings. Note. Midwives were asked what they themselves think should be reported, and what they think parents want to be reported, regarding secondary findings (SFs) and variants of uncertain significance (VUS). Response alternatives were “Yes”,” No”,” Do not know”

Respondents were presented with short texts explaining secondary findings (SFs) and uncertain findings and asked if they are familiar with these concepts, and if they have discussed the possibility of finding SFs with expecting parents. Most of the midwives stated that they were aware of SFs (*n* = 55, 78%), but fewer have discussed them with the parents (*n* = 15, 21%). Fewer midwives stated that they were aware of uncertain findings (*n* = 45, 63%), and even fewer had discussed them with parents (*n* = 15, 21%). Further, the respondents were asked whether they think SFs and uncertain findings should be reported to parents, and if they perceive that parents want this information reported (Fig. 3). For secondary findings, 62% (*n* = 44) of midwives prefer this to be reported, while 31% (*n* = 22) believe parents want this information. For uncertain findings 30% (*n* = 21) of midwives prefer this to be reported, while 23% (*n* = 16) perceive that parents want this to be reported. More than half of midwives indicated “I do not know” for these questions. Free-text comments include that these are difficult questions, and that it is difficult to not give information that is available. Ethical issues, for example that it can create unnecessary worry and that it depends on the type of condition what to inform, were also brought up. Several comments mention the importance of sufficient and accurate information before the test to set the right expectations.

## Discussion

The result of this study indicates that the majority of midwives discuss prenatal genetic testing, at some level, with most of the expecting parents that they meet. The most frequent topics discussed are the most established and well-known tests; with FCT and NIPT being the most frequently discussed methods, centering on well-known conditions. Aspects of other diagnostic methods and possible outcomes of invasive testing is indicated to be rarely discussed by primary maternity midwives with parents. When additional questions are asked by expecting parents, NIPT is frequently mentioned by midwives. The number of questions about NIPT stands out as being perceived to have increased markedly in the past three years, while midwives do not sense that the questions about sequencing and microarray methods have increased as much. This is in line with the introduction of NIPT, while the other methods are not yet well-known among parents or might not be relevant at that stage in the testing process. Lack of time and knowledge are not indicated to be significant barriers for midwives to discuss prenatal genetic testing. Midwives perceive that when questions are asked about specific conditions, they are commonly related to trisomy 21. This is likely because Downs syndrome is the most well-known condition among parents and therefore the main goal of prenatal testing for most parents. Interestingly, midwives indicated quite frequent questions about neuropsychiatric conditions, even though these conditions are not included in prenatal testing. This could reflect an increased awareness in the society and worry among parents for this type of conditions (SMER 2015).

The results of this study are in line with previous studies. According to the Swedish Pregnancy Register (2022) survey, most pregnant women in Sweden perceive they received adequate information after FCT test, but fewer before deciding about NIPT or invasive testing. An earlier survey study of Swedish midwives found that the amount of information given to pregnant women about prenatal diagnostics was less than would be needed for a complete understanding of relevant facts and risks. Despite this, pregnant women in the study were still overall satisfied with the amount of information and time spent (Ferm Widlund et al. 2009). More recently, a study found substantial variation in the amount of information and time given about screening methods and their possible outcomes to expecting parents at the antenatal clinic. There was also substantial variation in parents’ pre-existing knowledge and need for information. (Carlsson et al. 2021). Other studies have also found women’s informational needs to be variable when deciding about prenatal testing; some preferring a step-by step-process while others want extensive information from the start (Ternby et al. 2023). Midwives may need to inform expecting parents about things they do not themselves know to ask for, to ensure fully informed decisions. Furthermore, expecting parents may come with pre-existing views that may include misconceptions and misunderstandings (Lewis et al. 2016).

Ahead of implementation of NIPT it was highlighted that there is a risk that it is perceived as a routine test, and that clear and neutral information, as well as to reassure that the expecting parents have understood the information is essential (SBU 2015). Previous studies have found that different factors influence pregnant women´s decision making about NIPT; including how NIPT compares to other testing options, reflections on coping, and moral and religious values (Lewis et al. 2016). Thus, health providers need to have a good understanding of NIPT so that they can provide adequate pre-test counselling in this area. Recent studies focusing on clinical implementation of NIPT indicate that informed choice is possible to achieve for NIPT testing with adequate counselling and attention to personal factors such as education, language, and earlier pregnancies (Lewis et al. 2017; van der Meij et al. 2022). Pregnant women should ideally be met with personalized information and individualized counselling (Ternby et al. 2023). However, informational needs have also been found to not depend on sociodemographic background or coping style, making women’s variable information needs difficult to predict by healthcare professionals..

In this study, as could be expected, most midwives seem confident to answer the questions they meet frequently, and they feel confident that they have enough knowledge about FCT, which they also get asked most frequently about. A previous study indicated similar level of midwives´ confidence in knowledge about FCT (Ternby et al. 2015). The stated level of confidence in knowledge is lower for NIPT compared to FCT in this study, while the number of questions about NIPT is perceived as increasing. Midwives feel less knowledgeable about sequencing and microarray, but at the same time they receive a smaller number of questions about these methods, and this information is currently primarily given by fetal medicine specialists at a later stage in the process. While sequencing is still not a routine clinical method, microarray is, and a higher confidence could be expected for this method even for primary care midwives. In earlier studies, midwives have been found to have insufficient or no education about prenatal tests, and the knowledge was even less for conditions often screened for, such as Downs syndrome (Ternby et al. 2015).

Results from the survey indicate that many midwives are aware of uncertain findings and secondary findings that can arise with new sequencing methods, but fewer have discussed this with expecting parents. The reasons for this are likely that parents do not know to ask about this, or that midwives do not feel confident to have these discussions. Midwives are relatively more in favor of reporting secondary findings than uncertain findings. A study investigating 498 professionals` attitudes towards reporting variants of uncertain significance showed that 70% of them were in favor of reporting if the finding could be associated with the phenotype. For secondary findings, 93% wanted to report findings if treatment was available and 80% if this was not the case (Brew et al., 2019). On the other hand, the professionals studied were genetic counsellors, with probable more experience in the genetic field. The result from this study shows that the midwives do not have a strong perception whether the parents wish uncertain results to be reported or not. This is perhaps reflecting the complexity of the subject, and that few parents are even aware of these types of findings. Also, midwives themselves are more inclined to report secondary findings to the parents, than what they perceive the parents wish to receive those results. There is a high proportion of “I do not know”-answers in questions about uncertain and secondary findings. Previous studies have shown that prospective parents often want the maximum amount of information from an invasive test (Plantinga et al. 2022; van der Steen et al. 2015), including unexpected findings (Plantinga et al. 2022), and highly value being given the opportunity to choose the scope of testing themselves (van der Steen et al. 2015). Midwives, however, have in previous studies been found to be more concerned about uncertainties (Park et al. 2019). Previous study of health professionals’ views identified the importance of pre-test counselling to manage parents’ expectations. Pre-test counselling is suggested by the authors (Lewis, 2021) to also include information about the possibility to receive uncertain results. A standardization of competencies for midwives’ education such as in a competency framework (Tonkin et al. 2018), as well as national guidelines and recommendations regarding uncertain and secondary findings is desirable.

### Study limitations

This survey studies the midwives view, but does not reflect the expecting parents’ perception, or observe what happens when parents meet midwives. The study explores midwives perceived knowledge and confidence to answer questions, but does not measure their actual knowledge in an objective way. For a broader perspective, the results need to be put in context with observational studies, as well as studies of expecting parents’ views. The questionnaire was developed specifically for this study and has not been validated. The invitation to participate was distributed through a network of midwives and access to the questionnaire was online. Due to this the exact number of invitations is not known and it is not possible to measure exact response rate. There may have been selection bias as midwifes with higher interest may have responded to a higher degree. The smaller number of respondents makes comparison of distinct groups of limited use. As no significant differences were found between groups, no further statistical analysis was utilized. Higher number of respondents would have enabled more extensive comparison between different groups such as age, years in profession or location.

## Conclusions

Health providers need to have a good understanding of prenatal testing including conditions tested for. Ongoing education for midwives is essential - around established methods like NIPT, but also on more comprehensive genomic test methods such as microarray and massive parallel sequencing techniques, and challenges around test results, including uncertain results and secondary findings. This education should preferably occur prior to implementation of new methods in healthcare. It is important that midwives are being trained in pre-test counselling and that they are able to ensure that women receive individual counselling with personalized information, to support and enable informed decision making.

## Supplementary information

Below is the link to the electronic supplementary material.

Supplementary File 1 (DOCX 22.8 KB)

## Data Availability

No datasets were generated or analysed during the current study.

## Abbreviations

FCT: First Trimester Combined test
NIPT: Non-Invasive prenatal testing

## References

[CR1] Barnmorskeförbundet (2022) Retrieved from https://www.barnmorskeforbundet.se/

[CR2] Carlsson Y, Strömbäck P, Lundgren I (2021) Parents’ experiences of the information provided at the antenatal clinic regarding foetal diagnostics – a qualitative interview study. Sexual & Reproductive Healthcare 29:100652. 10.1016/j.srhc.2021.100652 34375881 10.1016/j.srhc.2021.100652

[CR3] Di Mattei V, Ferrari F, Perego G, Tobia V, Mauro F, Candiani M (2021) Decision-making factors in prenatal testing: a systematic review. Health Psychol Open 8(1):2055102920987455. 10.1177/2055102920987455 33489303 10.1177/2055102920987455 PMC7809316

[CR4] Dondorp W, de Wert G, Bombard Y, Bianchi DW, Bergmann C, Borry P (2015) American Society of Human Genetics, t. Non-invasive prenatal testing for aneuploidy and beyond: challenges of responsible innovation in prenatal screening. 23:1438–1450. Retrieved from https://login.e.bibl.liu.se/login?url=https://search.ebscohost.com/login.aspx?direct=true%26;AuthType=ip,uid%26;db=edsair%26;AN=edsair.doi.dedup.....32a6227a6287dc08a38e8fd952ead2dc%26;site=eds-live%26;scope=site. https://www.ncbi.nlm.nih.gov/pmc/articles/PMC4613463/pdf/ejhg201557a.pdf 10.1038/ejhg.2015.57 PMC4613463 25782669

[CR5] Etikprövningsmyndigheten (2022) Retrieved from https://etikprovningsmyndigheten.se/for-forskare/vad-sager-lagen/

[CR6] Ferm Widlund K, Gunnarsson C, Nordin K, Hansson MG (2009) Pregnant women are satisfied with the information they receive about prenatal diagnosis, but are their decisions well informed? Acta Obstet Gynecol Scand 88(10):1128–1132. 10.1080/00016340903144246 19642039 10.1080/00016340903144246

[CR7] God Forskningssed (2017) (Reviderad utgåva ed.). Stockholm: Vetenskapsrådet.

[CR8] Harding E, Hammond J, Chitty LS, Hill M, Lewis C (2020) Couples experiences of receiving uncertain results following prenatal microarray or exome sequencing: a mixed-methods systematic review. Prenat Diagn 40(8):1028–1039. 10.1002/pd.5729 32362033 10.1002/pd.5729 PMC8425413

[CR9] Harris S, Gilmore K, Hardisty E, Lyerly AD, Vora NL (2018) Ethical and counseling challenges in prenatal exome sequencing. Prenat Diagn 38(12):897–903. 10.1002/pd.5353 30171820 10.1002/pd.5353 PMC6370459

[CR10] Kernie CG, Wynn J, Rosenbaum A, de Voest J, Galloway S, Giordano J, Stover Samantha, Westerfield Lauren, Gilmore Kelly, Wapner Ronald J., den Van Veyver Ignatia B., Vora Neeta L., Clifton Rebecca G., Caughey Aaron B., Chung WK (2022) Information is power: the experiences, attitudes and needs of individuals who chose to have prenatal genomic sequencing for fetal anomalies. Prenat Diagn 42(7):947–954. 10.1002/pd.6153 35476893 10.1002/pd.6153 PMC11561471

[CR11] Klapwijk JE, Srebniak MI, Go A, Govaerts LCP, Lewis C, Hammond J, Riedijk SR (2021) How to deal with uncertainty in prenatal genomics: a systematic review of guidelines and policies. Clin Genet 100(6):647–658. 10.1111/cge.14010 34155632 10.1111/cge.14010 PMC8596644

[CR12] Lewis C, Hill M, Chitty LS (2016) A qualitative study looking at informed choice in the context of non-invasive prenatal testing for aneuploidy. Prenat Diagn 36(9):875–881. 10.1002/pd.4879 27477537 10.1002/pd.4879 PMC5053255

[CR13] Lewis C, Hill M, Chitty LS (2017) Offering non-invasive prenatal testing as part of routine clinical service. Can high levels of informed choice be maintained? Prenat Diagn 37(11):1130–1137. 10.1002/pd.5154 28892219 10.1002/pd.5154 PMC5969260

[CR14] Liu P, Vossaert L (2022) Emerging technologies for prenatal diagnosis: the application of whole genome and RNA sequencing. Prenat Diagn 42(6):686–696. 10.1002/pd.6146 35416301 10.1002/pd.6146 PMC10014115

[CR15] Lou S, Lomborg K, Lewis C, Riedijk S, Petersen OB, Vogel I (2020) It’s probably nothing, but… couples’ experiences of pregnancy following an uncertain prenatal genetic result. Acta Obstet Gynecol Scand 99(6):791–801. 10.1111/aogs.13813 31955407 10.1111/aogs.13813

[CR16] Mellis R, Oprych K, Scotchman E, Hill M, Chitty LS (2022) Diagnostic yield of exome sequencing for prenatal diagnosis of fetal structural anomalies: a systematic review and meta-analysis. Prenat Diagn 42(6):662–685. 10.1002/pd.6115 35170059 10.1002/pd.6115 PMC9325531

[CR17] Mellis R, Tapon D, Shannon N, Dempsey E, Pandya P, Chitty LS, Hill M (2022) Implementing a rapid fetal exome sequencing service: what do parents and health professionals think? Prenat Diagn 42(6):783–795. 10.1002/pd.6140 35383981 10.1002/pd.6140 PMC9324936

[CR18] Park J, Zayhowski K, Newson AJ, Ormond KE (2019) Genetic counselors’ perceptions of uncertainty in pretest counseling for genomic sequencing: a qualitative study. J Genet Couns 28(2):292–303. 10.1002/jgc4.1076 30741463 10.1002/jgc4.1076

[CR19] Plantinga M, Zwienenberg L, van Dijk E, Breet H, Diphoorn J, El Mecky J, Bouman Katelijne, Verheij Joke, Birnie Erwin, Ranchor Adelita V., Corsten‐Janssen Nicole, van Langen IM (2022) Parental experiences of rapid exome sequencing in cases with major ultrasound anomalies during pregnancy. Prenat Diagn 42(6):762–774. 10.1002/pd.6056 34643287 10.1002/pd.6056 PMC9298392

[CR20] Riksdagen (2006) The Genetic Integrity Act (2006:351). Retrieved from www.riksdagen.se/sv/dokument-lagar/dokument/svensk-forfattningssamling/lag-2006351-om-genetisk-integritet-mm_sfs-2006-351

[CR21] Riksdagen (2003) The Swedish Act (2003:460) concerning the ethical review of research involving humans. Retrieved from https://www.riksdagen.se/sv/dokument-lagar/dokument/svensk-forfattningssamling/lag-2003460-om-etikprovning-av-forskning-som_sfs-2003-460

[CR22] Sahin-Hodoglugil NN, Lianoglou BR, Ackerman S, Sparks TN, Norton ME (2023) Access to prenatal exome sequencing for fetal malformations: a qualitative landscape analysis in the US. Prenat Diagn 43(11):1394–1405. 10.1002/pd.6444 37752660 10.1002/pd.6444 PMC10846391

[CR23] SBU (2015) *Analys av foster-DNA i kvinnans blod: icke-invasiv fosterdiagnostik (NIPT) för trisomi 13, 18 och 21*. Retrieved from https://www.sbu.se/sv/publikationer/sbu-utvarderar/Analys-av-foster-DNA-i-kvinnans-blod-icke-invasiv-fosterdiagnostik-NIPT-for-trisomi-13-18-och-21/

[CR24] Skogsdal Y, Conner P, Elvander L, Storck Lindholm E, Kloow M, Algovik M, Granfors M (2023) *Graviditestegistrets Årsrapport 2022*. Retrieved from graviditetsregistret.se

[CR25] SMER (2012) Etisk analys för diagnostik med foster DNA. Retrieved from https://smer.se/wp-content/uploads/2012/05/Rapport-Fosterdiagnostik-Etisk-analys-for-diagnostik-med-foster-DNA.pdf

[CR26] SMER (2015) Analys av foster-DNA i kvinnans blod: Icke invasiv fosterdiagnostik (NIPT) för trisomi 13, 18 och 21 – etiska aspekter. Retrieved from https://smer.se/wp-content/uploads/2015/10/Smer-2015_1-webb-PDF.pdf

[CR27] Socialstyrelsens föreskrifter och allmänna råd om fosterdiagnostik och preimplantatorisk genetisk diagnostik (2012)

[CR28] *Sveriges Regioner i Samverkan*. (2022) Retrieved from https://d2flujgsl7escs.cloudfront.net/external/Fosterdiagnostik-kartlaggning-och-rekommendation.pdf

[CR29] Ternby E, Ingvoldstad C, Annerén G, Axelsson O (2015) Midwives and information on prenatal testing with focus on down syndrome. Prenat Diagn 35(12):1202–1207. 10.1002/pd.4676 26279318 10.1002/pd.4676

[CR30] Ternby E, Axelsson O, Georgsson S, Malmgren CI (2023) Pregnant women’s informational needs prior to decisions about prenatal diagnosis for chromosomal anomalies—A Q methodological study. Prenat Diagn n/a(n/a). 10.1002/pd.6514 10.1002/pd.6514 38167810

[CR31] Tonkin ET, Skirton H, Kirk M (2018) The first competency based framework in genetics/genomics specifically for midwifery education and practice. Nurse Educ Pract 33:133–140. 10.1016/j.nepr.2018.08.015 30296725 10.1016/j.nepr.2018.08.015

[CR32] van den Berg M, Timmermans DRM, ten Kate LP, van Vugt JMG, van der Wal G (2006) Informed decision making in the context of prenatal screening. Patient Educ Couns 63(1):110–117. 10.1016/j.pec.2005.09.007 16242899 10.1016/j.pec.2005.09.007

[CR33] van der Meij KRM, Njio A, Martin L, Gitsels-van der Wal JT, Bekker MN, van Vliet-Lachotzki EH, Henneman L (2022) Routinization of prenatal screening with the non-invasive prenatal test: pregnant women’s perspectives. Eur J Hum Genet 30(6):661–668. 10.1038/s41431-021-00940-8 34385671 10.1038/s41431-021-00940-8 PMC9177612

[CR34] van der Steen SL, Diderich KE, Riedijk SR, Verhagen-Visser J, Govaerts LC, Joosten M, Galjaard RJ (2015) Pregnant couples at increased risk for common aneuploidies choose maximal information from invasive genetic testing. Clin Genet 88(1):25–31. 10.1111/cge.12479 25134982 10.1111/cge.12479

[CR35] WMA (2013) World medical association declaration of helsinki: ethical principles for medical research involving human subjects. JAMA 310(20):2191–2194. 10.1001/jama.2013.281053 24141714 10.1001/jama.2013.281053

[CR36] Lewis C, Hammond J, Klapwijk JE, Harding E, Lou S, Vogel I, Szepe EJ, Hui L, Ingvoldstad-Malmgren C, Soller MJ, Ormond KE, Choolani M, Hill M, Riedijk S (2021) Dealing with uncertain results from chromosomal microarray and exome sequencing in the prenatal setting: An international cross-sectional study with healthcare professionals. Prenat Diagn. 41(6):720-732. doi: 10.1002/pd.5932 10.1002/pd.5932 PMC8519283 33724493

[CR37] Brew CE, Castro BA, Pan V, Hart A, Blumberg B, Wicklund C (2019) Genetics professionals' attitudes toward prenatal exome sequencing. J Genet Couns. 2019 Apr;28(2):229-239. doi: 10.1002/jgc4.1112 10.1002/jgc4.1112 30888706

