# Supplementary material for: Midwives ‘views of parents’ questions and expectations on prenatal genetic testing - identifying informational needs in prenatal genetic counselling
Source: J Community Genet. 2025 Dec 13;17(1):13. doi: 10.1007/s12687-025-00846-8 (PMC12701880; doi:10.1007/s12687-025-00846-8)
Supplement: Supplementary file 1 — (DOCX 22.8 KB) [file 12687_2025_846_MOESM1_ESM.docx]

Supplement 1. Questionnaire Midwives

**1. With how many of the expectant parents you meet in your work do you talk about genetic prenatal diagnostics?**

- 0-2 out of 10
- 3-4 out of 10
- 5-7 out of 10
- 8-10 out of 10
- Comment

**2. If you do not talk about genetic prenatal diagnostics with the expectant parents you meet, what is the most common reason?**

- Has not happened
- They have already received information in another way within maternal health care
- They have already received information from sources other than maternal health care
- The parents have chosen not to take part in this information
- I do not have sufficient knowledge
- Lack of time
- Other

**3. How many of the parents you meet ask questions beyond what is included in the regular information about genetic prenatal diagnostics?**

- 0-2 out of 10
- 3-4 out of 10
- 5-7 out of 10
- 8-10 out of 10
- What questions do you get? Please comment.

**4. How do you think the number of questions from expectant parents about genetic prenatal diagnostics has changed in the last 3 years? (not related to questions about changed availability during the pandemic)**

- Has decreased
- Unchanged
- Has increased
- Comment

**5. Do you feel that the age of expectant parents affects how much information they request about genetic prenatal diagnostics?**

- Yes, younger often ask more
- Yes, older often ask more
- I do not notice any difference
- Do not know
- Comment

**6. Do you feel that the educational level of expectant parents affects how much information they request about genetic prenatal diagnostics?**

- Yes, parents with lower education often ask more
- Yes, parents with higher education often ask more
- I do not notice any difference
- Do not know
- Comment

**7. In the offer of prenatal diagnostics, certain conditions are included. How common do you perceive the following expectations about the scope to be among expectant parents?**

- 1 - a few
- 2
- 3
- 4
- 5 - most
- Expect more conditions/diseases to be included than actually are
- 1
- 2
- 3
- 4
- 5
- Have realistic expectations
- 1
- 2
- 3
- 4
- 5
- Expect fewer conditions/diseases to be included than actually are
- 1
- 2
- 3
- 4
- 5
- Comment

**8. Have you received questions from expectant parents about prenatal tests for genetic predispositions for the following diseases or syndromes?**

- No, have not received
- Yes, occasionally-sometimes
- Yes, regularly-often
- Neuropsychiatric diagnoses
- x
- x
- x
- Psychiatric diseases
- x
- x
- x
- Diabetes
- x
- x
- x
- Cancer diseases
- x
- x
- x
- Trisomy 21, Down syndrome
- x
- x
- x
- Trisomy 18, 13 (Edwards, Patau syndrome)
- x
- x
- x
- **Sex chromosome abnormalities** (e.g., Turner's, Klinefelter's syndrome)
- x
- x
- x
- Microdeletion/microduplication syndromes
- x
- x
- x
- Other (please specify below)
- x
- x
- x
- Comment

**9. To what extent have you received questions from expectant parents about the following methods?**

- **Has this changed in the last 2 years?**
- **Never received**
- **Occasionally-sometimes**
- **Regularly-often**
- **Decreased**
- **Unchanged**
- **Increased**
- **KUB**
- **x**
- **x**
- **x**
- **x**
- **x**
- **x**
- **NIPT**
- **x**
- **x**
- **x**
- **x**
- **x**
- **x**
- **Microarray (analysis of invasive sample)**
- **x**
- **x**
- **x**
- **x**
- **x**
- **x**
- **Sequencing of the entire/parts of the fetus's genome (analysis of invasive sample)**
- **x**
- **x**
- **x**
- **x**
- **x**
- **x**
- **Comment**

**10. Do you feel that you have sufficient knowledge about the following prenatal tests/methods to be able to explain and answer questions from expectant parents in your work?**

- No
- Yes
- Partially
- NIPT
- x
- x
- x
- KUB
- x
- x
- x
- Microarray
- x
- x
- x
- Sequencing of the entire genome of the fetus
- x
- x
- x
- Comment

**11. There can be various reasons why expectant parents consider prenatal diagnostics. For parents who are interested in genetic prenatal diagnostics, how common do you perceive the following questions to be?**

- **1 - not common**
- **2**
- **3**
- **4**
- **5 - very common**
- **Test for a specific hereditary disease that exists in the family/relatives**
- **1**
- **2**
- **3**
- **4**
- **5**
- **Test for various rare, serious diseases**
- **1**
- **2**
- **3**
- **4**
- **5**
- **Test the risk of developing a common disease in adulthood**
- **1**
- **2**
- **3**
- **4**
- **5**
- **Test for as much as possible; "know that everything is fine" with the fetus**
- **1**
- **2**
- **3**
- **4**
- **5**
- **Do you encounter other questions? Please describe.**

**The following questions concern invasive tests, i.e., chorionic villus sampling or amniocentesis analyzed with some genetic analysis method. Answer based on your own opinion. There are no "right" or "wrong" answers.**

**12. Incidental findings: Sometimes prenatal tests can find genetic variants that are not related to the condition being investigated (e.g., an abnormality detected on ultrasound) but are still of medical significance. These can be called incidental findings (secondary findings and incidental findings). These can be reported or not reported to parents.**

- No
- Yes
- Do not know
- Are you aware of genetic incidental findings from before?
- x
- x
- x
- Have you discussed the possibility of genetic incidental findings with expectant parents?
- x
- x
- x
- Do you feel that parents generally want genetic incidental findings reported?
- x
- x
- x
- Do you think that genetic incidental findings should be reported to parents?
- x
- x
- x
- Comment

**13. Uncertain findings: Sometimes prenatal tests can show genetic variants that cannot be definitively said to be disease-causing or not. It can be difficult or impossible even for doctors to predict if and how such variants may affect the child. This type of uncertain result can be reported or not reported to parents.**

- No
- Yes
- Do not know
- Are you aware of uncertain results from before?
- x
- x
- x
- Have you discussed the possibility of uncertain results with expectant parents?
- x
- x
- x
- Do you feel that parents generally want reporting of uncertain results?
- x
- x
- x
- Do you think that uncertain results should be reported to parents?
- x
- x
- x
- Comment

**14. Different tests can have different advantages and disadvantages that can affect the choice. How important do you think a typical parent considers the following aspects when deciding on an invasive genetic prenatal diagnostic test (on a scale of 1 (not so relevant) - 5 (very relevant)):**

- Who provides the parents with information about the test offer (midwife or specialist)
- Risk of miscarriage related to the invasive test
- The likelihood that the test actually finds a disease-causing genetic abnormality
- Time to get the test result
- Whether the test provides information about incidental findings (not related to the original reason for the test)
- Whether all genetic abnormalities found in the fetus are reported – even uncertain results
- Comment

Below are demographic questions:

**15. Which age group do you belong to?**

- under 35 years
- 35-50 years
- over 50 years

**16. How long have you worked as a midwife?**

- 0-4 years
- 5-15 years
- 16-25 years
- over 25 years

**17. Which statement best describes your professional role?**

- Midwife in primary maternal health care
- Ultrasound midwife
- Other

**18. What type of clinic do you work at?**

- Private
- Public
- Other

**19. What type of city/town is the clinic you work at located in?**

- Big city
- Suburb of a big city
- Medium-sized city/town
- Small city/town
- Other

**20. In which region do you work?**

- **Northern healthcare region**
- **Central Sweden healthcare region**
- **Stockholm healthcare region**
- **Southeastern healthcare region**
- **Western healthcare region**
- **Southern healthcare**
